# Cardiovascular Health Score and Atherosclerotic Cardiovascular Disease in the Million Veteran Program

**DOI:** 10.1001/jamanetworkopen.2024.47902

**Published:** 2024-12-06

**Authors:** Xuan-Mai T. Nguyen, Yanping Li, Yusi Gong, Serena Houghton, Yuk-Lam Ho, Mary Pyatt, Timothy Treu, Ruifeng Li, Kitan Akinosho, Sridharan Raghavan, David R. Gagnon, John Michael Gaziano, Peter W. F. Wilson, Kelly Cho

**Affiliations:** 1 Million Veteran Program Boston Coordinating Center, VA Boston Healthcare System, Boston, Massachusetts; 2 Department of Medicine, UCLA David Geffen School of Medicine, Los Angeles, California; 3 Department of Nutrition, Harvard T. H. Chan School of Public Health, Boston, Massachusetts; 4 Beth Israel Deaconess Medical Center, Boston, Massachusetts; 5 Department of Veterans Affairs Eastern Colorado Healthcare System, Aurora; 6 Boston University School of Public Health, Boston, Massachusetts; 7 Division of Aging, Brigham and Women′s Hospital, Boston, Massachusetts; 8 Department of Medicine, Harvard Medical School, Boston, Massachusetts; 9 Department of Epidemiology, Rollins School of Public Health, Emory University, Atlanta, Georgia; 10 Atlanta VA Health Care System, Decatur, Georgia; 11 Emory Clinical Cardiovascular Research Institute, Atlanta, Georgia

## Abstract

**Importance:**

The American Heart Association proposed Life’s Essential 8 (LE8) as an enhanced measurement tool for cardiovascular health.

**Objective:**

To examine the association of LE8 with risk of atherosclerotic cardiovascular disease (ASCVD) incidence and prognosis in veterans.

**Design, Setting, and Participants:**

This was a prospective cohort study of US veterans enrolled in the Department of Veterans Affairs (VA) Million Veteran Program (MVP) between 2011 and 2022. Data were analyzed from 2023 to 2024.

**Exposure:**

LE8 score ranged from 0 to 100, with higher score indicating better cardiovascular health.

**Main Outcomes and Measures:**

The primary outcome was total ASCVD incidence in veterans without baseline ASCVD, and the secondary outcome was incidence of a major adverse cardiovascular event (MACE) among veterans with and without ASCVD at baseline.

**Results:**

A total of 413 052 veterans (mean [SD] age, 65.8 [12.1] years; 378 162 [91.6%] male) were included. Based on 1.7 million person-years of follow-up of 279 868 veterans without any ASCVD at baseline, 45 067 veterans had an ASCVD event during follow-up. Total LE8 score and each component LE8 factor score was associated with incident ASCVD in an inverse, linear, dose-response manner. For veterans without prior ASCVD, those with an LE8 score between 80 and 100 had lower risk of ASCVD compared with those with an LE8 score of 0 to 49 (adjusted hazard ratio [aHR], 0.36 [95% CI, 0.35-0.38]). Similarly, risk of MACE was significantly lower among veterans with an LE8 score of 80 to 100 regardless of baseline ASCVD status (with ASCVD: aHR, 0.52 [95% CI, 0.48-0.56]; without ASCVD: aHR, 0.14 [95% CI, 0.13-0.15]) compared with those with ASCVD and an LE8 score of 0 to 49.

**Conclusions and Relevance:**

In this cohort study of US veterans, higher LE8 scores were associated with significantly lower ASCVD incidence risk and lower likelihood of developing adverse cardiovascular events regardless of ASCVD status at baseline. These results support the utility of LE8 for health promotion and ASCVD prevention.

## Key Points

**Question:**

What is the association of Life’s Essential 8 (LE8), an enhanced measurement tool for cardiovascular health, with atherosclerotic cardiovascular disease (ASCVD) in veterans?

**Findings:**

In a cohort study including 413 052 veterans with 2.7 million person-years of follow-up, higher uptake of LE8 components was associated with significantly lower ASCVD incidence, likelihood of developing major adverse cardiovascular events, and incidence of all-cause mortality.

**Meaning:**

These findings suggest that a healthy lifestyle aimed at promoting ideal cardiovascular health, such as LE8, plays an important role not only in the primary prevention of ASCVD but also in the prevention of major adverse events after diagnosis of ASCVD.

## Teaser

This cohort study examines the association of a cardiovascular health score, Life’s Essential 8, with risk of atherosclerotic cardiovascular disease and other cardiovascular outcomes in veterans enrolled in the Million Veteran Program

## Introduction

Atherosclerotic cardiovascular disease (ASCVD) is the leading cause of mortality and disability in the US.^1,2,3^ Approximately 26 million adults live with ASCVD in the US, contributing to 2 million hospitalizations and 400 000 deaths every year.^2,3,4^ Adults with preexisting ASCVD are often at a higher risk for recurrent cardiovascular events.^4,5,6,7,8^ For example, individuals with peripheral arterial disease (PAD) or carotid artery disease have a 2- to 3-fold higher risk for stroke or transient ischemic attack^6^ and a 6-fold higher risk for CVD mortality compared with individuals without,^7^ and persons with any ASCVD had a 3- to 6-fold higher risk for a major adverse cardiovascular event (MACE).^8^ Prevention of ASCVD is a public health priority, and lifestyle modification is at the cornerstone for both primary and secondary prevention of ASCVD.^9,10^

The American Heart Association’s Life’s Essential 8 (LE8) is an updated construct of cardiovascular health based on modifiable lifestyle behaviors in addition to clinical health factors. These include a healthy diet, staying physically active, avoiding nicotine exposure, maintaining sleep health, managing a healthy body mass index (BMI), blood lipid control, blood glucose control, and blood pressure control as part of 8 criteria.^10^ Studies show a strong inverse association between LE8 score and risk of coronary heart disease (CHD),^11,12^ stroke,^11,12,13^ CVD death, all-cause mortality,^14,15,16,17,18^ and reduced life expectancy.^18,19^

It remains unclear whether LE8 is associated with comprehensive ASCVD incidence among US veterans. We aim to add to the evidence for LE8 score and its influence on first and subsequent incident ASCVD. Additionally, to our knowledge, no study to date has quantified the association between LE8 and incident MACE among individuals with preexisting ASCVD. Leveraging the expansive data from the Million Veteran Program (MVP),^20,21^ this study estimates the association of LE8 with risk of ASCVD incidence and MACE.

## Methods

This cohort study was approved by the Department of Veterans Affairs (VA) central institutional review board.^14,15^ All participants signed informed consent. This study is reported following the Strengthening the Reporting of Observational Studies in Epidemiology (STROBE) reporting guideline.

### Study Population

The VA Million Veteran Program (MVP) is a nationally representative, prospective cohort study of veterans designed to examine genetic and nongenetic factors associated with chronic diseases. MVP combines data from self-reported surveys (MVP Baseline and Lifestyle Surveys), electronic health records (EHR), and biospecimen samples collected at enrollment. Details of the research design can be found elsewhere.^20,21^ Enrollment of MVP participants began in 2011, with 913 319 veterans enrolled as of September 2022.

We included MVP enrollees who completed the MVP Lifestyle Survey, which includes information collected on socioeconomic status and lifestyle factors at baseline. Additional information on health conditions, comorbidities, and medication use was obtained through the Veterans Health Administration EHR. We excluded those with implausible death records and participants with missing data on 3 or more LE8 factors. We further split the cohort into those with and without ASCVD at baseline to evaluate incident ASCVD in participants without ASCVD at baseline and incident MACE in both cohorts.

### Assessment of the LE8

The LE8 score includes 8 components of cardiovascular health: healthy diet, staying physically active, avoiding nicotine exposure, maintaining sleep health, managing a healthy BMI, blood lipid control, blood glucose control, and blood pressure control.^10^ To evaluate healthy diet, dietary information was collected at baseline with an extensively validated semiquantitative food frequency questionnaire asking about the frequency with which the participant had consumed a specific portion size for 61 food items over the preceding year.^22,23^ Responses were converted to mean daily intake for each participant. Overall dietary quality for MVP participants was determined by using a Dietary Approaches to Stop Hypertension score based on intake of 8 components: fruits, vegetables, nuts and legumes, low-fat dairy products, whole grains, sugar-sweetened beverages, red and processed meat, and energy-adjusted sodium.^24,25^ Participant physical activity was measured via a survey regarding the frequency of moderate (ie, causing slightly increased heart rate) and vigorous (ie, causing heart to beat rapidly and breathing heavily) activity while at work, while performing chores in and around the home, and during leisure or free time. The total minutes of moderate (or greater) intensity activities per week were the sum of all moderate and vigorous activities at work, home, and leisure time.^26^ Sleep health was assessed via self-report. Participants were asked to report “How many hours do you usually sleep each day (24-hour period)?” with 6 prespecified responses: 5, 6, 7, 8, 9, and 10 or more hours.^27^ Smoking status was determined using an algorithm developed with EHRs in combination with self-reported data that classify individuals as current, former, or never smokers.^28^ Second-hand smoke exposure was self-reported in the MVP Baseline Survey and was defined as exposure to cigarette smoke at primary residence and not directly smoked by the individual. As we did not collect information regarding years since smoking cessation, we applied the total years of smoking among ever smokers as a surrogate. BMI was calculated from body weight and height data self-reported from the MVP Baseline Survey and corroborated by EHR data. Hemoglobin A_1c_ (HbA_1c_) levels were obtained from EHR data, and the measure closest to enrollment date was used to assess blood glucose, since fasting status for blood glucose measurements was not consistently known for participants. A diagnosis of type 2 diabetes was defined as having either at least 1 *International Classification of Diseases, Ninth Revision *(*ICD-9*) code 250.xx or *International Statistical Classification of Diseases and Related Health Problems, Tenth Revision *(*ICD-10*) for 2015 onwards (*ICD-10* code E08.x, E09.x, E10.x, E11.x, E13.x) at a primary care practitioner visit or at least 2 uses of the code in any setting and having an outpatient prescription for a diabetes drug (based on Veterans Health Administration national drug codes).^29,30,31^ Blood lipid control was assessed using total and high-density lipoprotein (HDL) cholesterol measurements derived from clinical laboratory data that were adjudicated by a panel of clinicians reviewing laboratory values across all VA locations. Finally, blood pressure control was assessed using systolic and diastolic blood pressure data derived from the EHR data closest to enrollment date.

Detailed information regarding scoring and cutoff points for each factor is presented in eTable 1 in Supplement 1. An overall LE8 score was calculated for each study participant using the mean of the 8 metrics. Final scores ranged from 0 to 100, and as suggested by the American Heart Association LE8 writing group, achieving an overall LE8 score of 80 to 100 was considered high cardiovascular health (CVH) and 0 to 49 was defined as low CVH.^10^

For participants missing 1 or 2 LE8 factors, we calculated the LE8 score based on the mean of available metrics. We conducted a subsequent sensitivity analysis to examine only participants with full data for all 8 LE8 factors and another sensitivity analysis among participants with any LE8 data (regardless of how many metrics were missing), with the LE8 score based on the mean of available metrics.

### Outcome Ascertainment

Among veterans without ASCVD at baseline, ASCVD incidence and all-cause mortality were identified through combined information from the VA Corporate Data Warehouse,^32,33^ which is the VA equivalent of an EHR system, Centers for Medicaid & Medicare Services database, and National Death Index database using *ICD-9* and *ICD-10* codes (eTable 2 in Supplement 1). Veterans were classified as having an individual ASCVD event if they had at least 1 diagnosis as an inpatient or at least 2 diagnoses as an outpatient within the follow-up period. Composite ASCVD events^34,35^ included any nonfatal acute coronary syndrome (including unstable angina and myocardial infarction [MI] with or without coronary revascularization), coronary artery disease, nonfatal stroke (including transient ischemic attack, ischemic stroke, and cerebral revascularization), CVD death, and PAD. Among veterans with and without ASCVD at baseline, we calculated a secondary end point of incident MACE,^36,37^ defined as experiencing a nonfatal stroke (either ischemic or hemorrhagic), nonfatal MI, or CVD death.

### Assessment of Covariates

Baseline data on age, sex, race, ethnicity, statin use, BMI, and baseline comorbidities were cross-referenced with the EHR, and remaining covariates were taken from the self-reported MVP Baseline Survey. Participant race and ethnicity were self-reported, and data were obtained from the MVP Baseline Survey or EHR. Hispanic was defined as answering yes to the MVP Baseline Survey question “Are you Spanish, Hispanic, or Latino?” or classification as Hispanic or Latinx in EHR. Race was defined by self-reported response to “What is your race?” on the MVP Baseline Survey. If race was not selected as Black or White, it was considered other, which included American Indian or Alaska Native, Asian Indian, Chinese, Filipino, Japanese, other Asian, and other Pacific Islander on the MVP Baseline Survey or EHR. Race and ethnicity were assessed to analyze whether differences between cardiovascular health metrics and ASCVD risk differed by racial or ethnic groups.

### Statistical Analysis

We defined baseline as the time a participant completed the MVP Lifestyle Survey. For those without ASCVD at baseline, we defined the end of follow-up as either the first diagnosis of ASCVD or MACE, death, or September 30, 2023, whichever came first. For those with ASCVD at baseline, we defined end of follow-up as the first incident of MACE after the enrollment period, death, or September 30, 2023, whichever came first.

A Cox proportional hazards model was used to estimate hazard ratios (HRs) and 95% CIs of ASCVD and its subtypes according to LE8 score. We first generated crude models then adjusted for age (continuous), sex (male or female), race and ethnicity (Hispanic, non-Hispanic Black, non-Hispanic White, and other), education level (≤high school or General Educational Development, some college, or ≥college), income level (<$30 000, $30 000-$59 000, ≥$60 000, or missing), marital status (currently married: yes, no, or missing), family history of cardiovascular disease (yes or no), and history of ever being diagnosed with atrial fibrillation, heart failure, cancer (except nonmelanoma skin cancer) at or before the time of the MVP Lifestyle Survey completion. We stratified the main analysis by sex, age, race, and ethnicity, and tested the multiplicative interaction between these factors and LE8 by comparing the −2 log likelihood of the multivariate adjusted models with and without the cross-product interaction term.^38^ All data analyses were performed using SAS software version 9.4 (SAS Institute), and statistical significance was set at 2-sided *P* < .05.

In a secondary analysis, we estimated the joint effects of ASCVD and LE8 score on MACE and total mortality. Among participants without ASCVD or with ASCVD but not MACE at baseline, we defined the incident MACE as the first ever diagnosis of MI, stroke, or fatal CVD. For participants ever diagnosed with MI or stroke at baseline, incident MACE was the first recurrent MI, stroke, or fatal CVD after baseline. In addition to the aforementioned covariates for ASCVD incidence, we further adjusted duration from the first diagnosis of ASCVD to baseline enrollment (which was set to zero for participants without ASCVD at baseline) and use of medications for cardiovascular diseases at baseline.

To assess the robustness of our estimates, we did several sensitivity analyses. First, we included only the participants who had complete data for all 8 LE8 metrics. Second, we excluded participants with a follow-up period less than 1 year and incident events within the first year of follow-up to address potential reverse causality. Third, to further address the potential for reverse causality, we conducted a sensitivity analysis among a relatively healthy population, excluding participants with diagnosis of atrial fibrillation, heart failure, or cancers at or before baseline. Fourth, we excluded the participants with no further medical visits after baseline to address concerns for loss-to-follow-up. Fifth, we included all the participants who had incomplete data on 3 to 7 of the 8 LE8 metrics. Finally, we applied a competing-risk regression model for different subtypes of ASCVD by including LE8 score as exposure and other risk factors as unconstrained covariates, allowing the effects of the covariates to vary across ASCVD subtypes.^39^ Data were analyzed from 2023 to 2024.

## Results

Of 913 319 MVP enrollees, 417 727 participants (45.7%) completed the MVP Lifestyle Survey (eTable 3 in Supplement 1). Of these 417 727 veterans, 901 (0.2%) were excluded due to implausible death records and 3774 (0.9%) were excluded due to missing data for 3 or more LE8 factors. The final study cohort included 413 052 participants (mean [SD] age, 65.8 [12.1] years; 378 162 [91.6%] male), with 26 571 Hispanic participants (6.4%), 44 777 non-Hispanic Black participants (10.8%), 329 365 non-Hispanic White participants (79.7%), and 12 339 participants (3.0%) of other race or ethnicity. At baseline, participants with higher LE8 scores (high CVH) were more likely to be married, non-Hispanic White females with fewer baseline comorbidities and with a higher level of education and family income (Table 1).

**Table 1.** Baseline Characteristics According to LE8 Score^a^

Characteristic	LE8 Score, No. (%)								
	0-49 (Low CVH) (n = 95 482)	50-54 (n = 51 333)	55-69 (n = 57 798)	60-64 (n = 57 089)	65-69 (n = 50 882)	70-74 (n = 40 407)	75-79 (n = 28 481)	80-100 (High CVH) (n = 31 580)	All (N = 413 052)
Age, mean (SD), y	64.7 (10.3)	65.9 (11.2)	66.2 (11.6)	66.5 (12.1)	66.7 (12.6)	66.4 (13.2)	66.1 (13.7)	64.2 (15.1)	65.8 (12.1)
Sex									
Female	6680 (7.0)	3614 (7.0)	4235 (7.3)	4333 (7.6)	4159 (8.2)	3784 (9.4)	3079 (10.8)	5006 (15.9)	34 890 (8.5)
Male	88 802 (93.0)	47 719 (93.0)	53 563 (92.7)	52 756 (92.4)	46 723 (91.8)	36 623 (90.6)	25 402 (89.2)	26 574 (84.2)	378 162 (91.6)
Race and ethnicity									
Hispanic	6422 (6.7)	3501 (6.8)	3893 (6.7)	3652 (6.4)	3211 (6.3)	2423 (6.0)	1675 (5.9)	1794 (5.7)	26 571 (6.4)
Non-Hispanic Black	14 973 (15.7)	6714 (13.1)	6658 (11.5)	5822 (10.2)	4376 (8.6)	2971 (7.4)	1820 (6.4)	1443 (4.6)	44 777 (10.8)
Non-Hispanic White	71 165 (74.5)	39 582 (77.1)	45 602 (78.9)	45 876 (80.4)	41 779 (82.1)	33 811 (83.7)	24 140 (84.8)	27 410 (86.8)	329 365 (79.7)
Other^b^	2922 (3.1)	1536 (3.0)	1645 (2.8)	1739 (3.0)	1516 (3.0)	1202 (3.0)	846 (3.0)	933 (3.0)	12 339 (3.0)
Education level									
<High school	3970 (5.0)	1763 (4.0)	1648 (3.3)	1394 (2.8)	1001 (2.2)	625 (1.7)	326 (1.3)	195 (0.7)	10 922 (3.0)
High school	21 949 (27.7)	10 833 (24.6)	11 446 (22.8)	10 293 (20.5)	8049 (17.7)	5503 (15.1)	3181 (12.3)	2348 (8.0)	73 602 (20.4)
≥Some college	53 416 (67.3)	31 355 (71.3)	37 161 (73.9)	38 495 (76.7)	36 326 (80.1)	30 300 (83.2)	22 424 (86.5)	26 658 (91.3)	276 135 (76.6)
Annual family income, $									
<30 000	30 982 (42.7)	14 760 (36.9)	15 500 (33.9)	14 021 (30.8)	11 499 (28.1)	8027 (24.5)	4999 (21.5)	4636 (17.8)	104 424 (32.0)
30 000-59 000	25 790 (35.6)	14 705 (36.8)	16 592 (36.3)	16 360 (36.0)	14 554 (35.5)	11 227 (34.3)	7661 (32.9)	7532 (29.0)	114 421 (35.0)
≥60 000	15 717 (21.7)	10 487 (26.2)	13 566 (29.7)	15 126 (33.2)	14 908 (36.4)	13 443 (41.1)	10 619 (45.6)	13 816 (53.2)	107 682 (33.0)
Currently married	43 469 (55.1)	26 044 (59.6)	30 724 (61.5)	31 469 (63.1)	29 356 (65.1)	23 993 (66.3)	17 398 (67.5)	19 697 (68.0)	222 150 (62.0)
Family history of CVD	32 197 (33.7)	17 993 (35.1)	20 703 (35.8)	20 795 (36.4)	18 903 (37.2)	15 391 (38.1)	11 205 (39.3)	12 825 (40.6)	150 012 (36.3)
Diseases at or before baseline									
Atrial fibrillation	11 566 (12.1)	5771 (11.2)	6312 (10.9)	5816 (10.2)	4931 (9.7)	3503 (8.7)	2276 (8.0)	2049 (6.5)	42 224 (10.1)
Heart failure	13 241 (13.9)	4996 (9.7)	4672 (8.1)	3700 (6.5)	2660 (5.2)	1603 (4.0)	948 (3.3)	650 (2.1)	32 470 (7.8)
Cancer	58 698 (61.5)	29 545 (57.6)	32 233 (55.8)	30 428 (53.3)	25 839 (50.8)	19 393 (48.0)	12 702 (44.6)	12 234 (38.7)	221 072 (53.0)

Abbreviations: CVD, cardiovascular disease; CVH, cardiovascular health; LE8, Life’s Essential 8.

^a^ Values are means or percentages and are standardized to the age distribution of the study population except age.

^b^ Other includes participants who responded “No, not Spanish, Hispanic, Latino” and any combination of the following: American Indian or Alaska Native, Asian Indian, Chinese, Filipino, Japanese, other Asian, Pacific Islander, or other.

### Incident ASCVD Among Veterans Without ASCVD at Baseline

Among 279 868 veterans without a history of ASCVD at baseline, 45 067 (16.1%) developed ASCVD during 1 719 512 person-years of follow-up. The incidence rate of ASCVD was 3.91 per 100 person-years among veterans with low CVH and 1.31 per 100 person-years among veterans with high CVH, with a graded decrease in incidence with higher LE8 score (Table 2). Compared with participants with low CVH, hazard of ASCVD was significantly lower for veterans with high CVH (adjusted HR [aHR], 0.36 [95% CI, 0.35-0.38]). We observed an overall trend showing a graded decrease in hazard of ASCVD with higher LE8 score (Table 2).

**Table 2.** Hazard of Atherosclerotic Cardiovascular Disease According to LE8 Score Among Million Veteran Program Participants Without Atherosclerotic Cardiovascular Disease at Baseline (N = 279 868)

Outcome	LE8 Score, estimate (95% CI)								*P* value for trend	HR per 10 LE8 (n = 279 868)^a^
	0-49 (n = 53 440)	50-54 (n = 32 043)	55-59 (n = 38 176)	60-64 (n = 39 746)	65-69 (n = 36 924)	70-74 (n = 30 614)	75-79 (n = 22 537)	80-100 (n = 26 658)		
Events, No. (%)	12 239 (22.9)	6120 (19.1)	6576 (17.2)	6333 (15.9)	5148 (13.9)	3924 (12.8)	2513 (11.2)	2214 (8.3)	NA	45 067 (16.1)
Person-years	312 827	193 362	232 860	243 180	231 182	193 777	142 900	169 424	NA	1 719 512
IR	3.91 (3.84- 3.98)	3.17 (3.09-3.25)	2.82 (2.76- 2.89)	2.60 (2.54-2.67)	2.23 (2.17-2.29)	2.03 (1.96-2.09)	1.76 (1.69-1.83)	1.31 (1.25-1.36)	NA	2.62 (2.60-2.65)
Crude HR	1 [Reference]	0.81 (0.79-0.84)	0.72 (0.70-0.75)	0.67 (0.65-0.69)	0.57 (0.55-0.59)	0.52 (0.50-0.54)	0.45 (0.43-0.47)	0.34 (0.32-0.35)	<.001	0.78 (0.77-0.79)
Adjusted HR^a^	1 [Reference]	0.79 (0.77-0.82)	0.70 (0.68-0.72)	0.65 (0.63-0.67)	0.55 (0.53-0.57)	0.51 (0.50-0.53)	0.45 (0.43-0.47)	0.36 (0.35-0.38)	<.001	0.78 (0.78-0.79)
Adjusted HR by sex^a^^,^^b^										
Male (n = 249 770)	1 [Reference]	0.80 (0.77-0.82)	0.71 (0.69-0.73)	0.65 (0.63-0.67)	0.56 (0.54-0.57)	0.52 (0.50-0.54)	0.46 (0.44-0.48)	0.37 (0.35-0.39)	<.001	0.79 (0.78-0.79)
Female (n = 30 098)	1 [Reference]	0.72 (0.63-0.83)	0.66 (0.58-0.76)	0.59 (0.52-0.68)	0.52 (0.45-0.60)	0.46 (0.39-0.55)	0.40 (0.33-0.48)	0.31 (0.26-0.37)	<.001	0.75 (0.73-0.78)
Adjusted HR by baseline age, y^a^^,^^c^										
<66 (n = 143 494)	1 [Reference]	0.81 (0.77-0.84)	0.71 (0.67-0.74)	0.65 (0.62-0.68)	0.54 (0.51-0.57)	0.47 (0.44-0.50)	0.41 (0.38-0.44)	0.29 (0.26-0.31)	<.001	0.76 (0.75-0.77)
≥66 (n = 136 374)	1 [Reference]	0.83 (0.79-0.87)	0.76 (0.73-0.79)	0.72 (0.69-0.75)	0.63 (0.60-0.65)	0.61 (0.58-0.64)	0.54 (0.51-0.57)	0.45 (0.43-0.48)	<.001	0.83 (0.82-0.84)
Adjusted HR by race and ethnicity^a^^,^^d^										
Hispanic (n = 19 716)	1 [Reference]	0.70 (0.58-0.85)	0.64 (0.52-0.77)	0.59 (0.49-0.72)	0.48 (0.39-0.59)	0.45 (0.35-0.57)	0.36 (0.27-0.48)	0.30 (0.22-0.41)	<.001	0.74 (0.71-0.78)
Non-Hispanic Black (n = 32 332)^a^	1 [Reference]	0.77 (0.71-0.83)	0.73 (0.67-0.79)	0.67 (0.61-0.73)	0.56 (0.50-0.62)	0.46 (0.41-0.53)	0.46 (0.39-0.55)	0.38 (0.30-0.47)	<.001	0.78 (0.76-0.80)
Non-Hispanic White (n = 218 726)^a^	1 [Reference]	0.80 (0.78-0.83)	0.71 (0.68-0.73)	0.65 (0.63-0.67)	0.56 (0.54-0.58)	0.53 (0.51-0.55)	0.46 (0.44-0.48)	0.37 (0.35-0.39)	<.001	0.79 (0.78-0.79)
Other (n = 9094)^e^	1 [Reference]	0.70 (0.57-0.85)	0.61 (0.50-0.74)	0.59 (0.49-0.72)	0.49 (0.39-0.60)	0.47 (0.37-0.59)	0.39 (0.27-0.50)	0.37 (0.27-0.50)	<.001	0.76 (0.72-0.81)

Abbreviations: HR, hazard ratio; IR, incidence rate; LE8, Life’s Essential 8; NA, not applicable.

^a^ Adjusted for age (continuous), sex (male or female), race and ethnicity (Hispanic, non-Hispanic Black, non-Hispanic White, and other), education level (≤high school or General Educational Development, some college, or ≥college), income level (<$30 000, $30 000-$59 000, ≥$60 000, or missing), marriage status (currently married: yes, no, or missing), family history of cardiovascular disease (yes or no), and diagnosis of atrial fibrillation, heart failure, or cancer (excluding nonmelanoma skin cancer) at or before baseline, except for the stratified indicator.

^b^ *P* for interaction = .08.

^c^ *P* for interaction <.001.

^d^ *P* for interaction = .98

^e^ Other includes participants who responded “No, not Spanish, Hispanic, Latino” and any combination of the following: American Indian or Alaska Native, Asian Indian, Chinese, Japanese, Filipino, other Asian, Pacific Islander, or other.

There was no difference in associations of LE8 with ASCVD between males and females or across race and ethnicity groups (Table 2). The association was significantly stronger among veterans younger than 66 years (aHR, 0.29 [95% CI, 0.26-0.31]) compared with those 66 years or older (aHR, 0.45 [95% CI, 0.43-0.48]) (*P* for interaction < .001).

### MACE and All-Cause Mortality Associated With Baseline ASCVD and LE8

**Figure 1.**
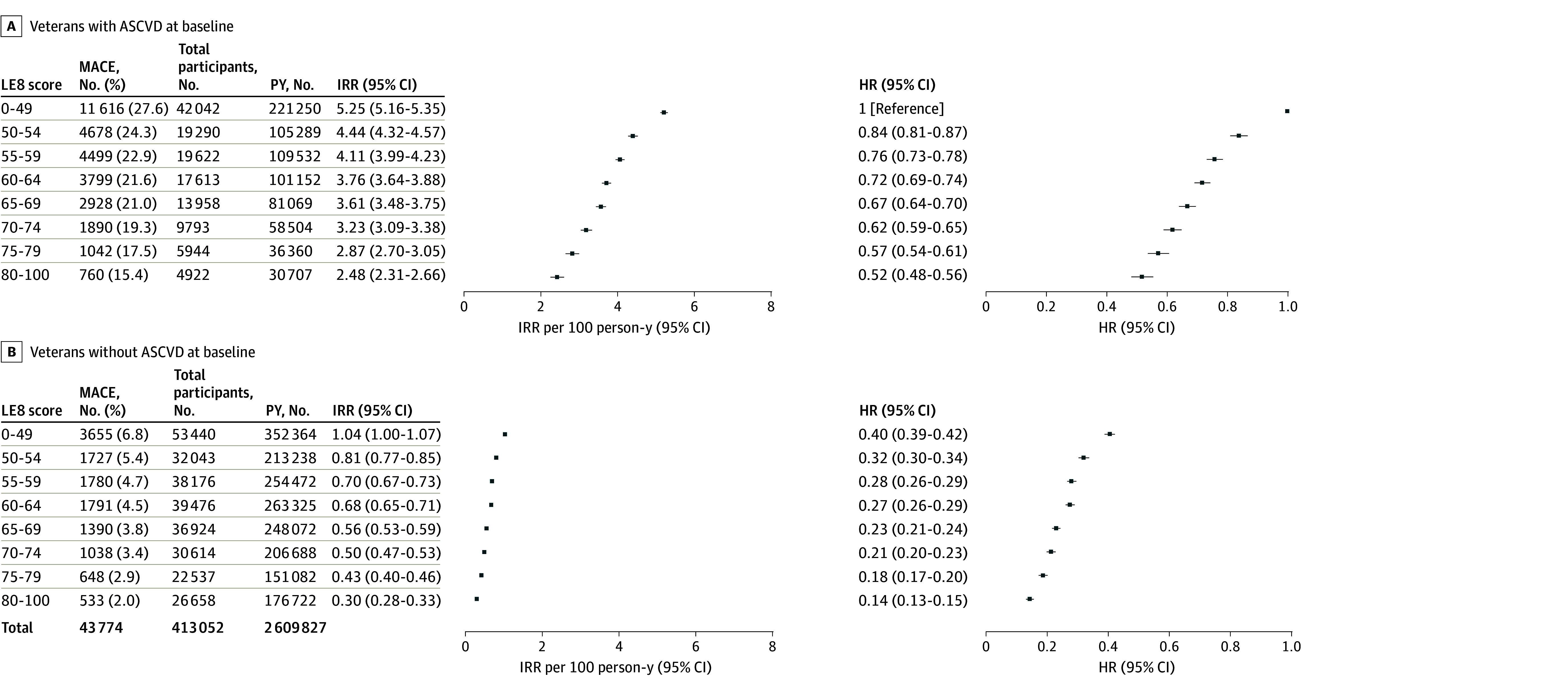
Incident Rates and Hazard of Major Adverse Cardiovascular Events (MACE) Among Veterans With and Without Atherosclerotic Cardiovascular Disease (ASCVD) at Baseline MACE were defined as experiencing a nonfatal stroke (either ischemic or hemorrhagic), nonfatal myocardial infarction, or cardiovascular disease death. Hazard ratios (HRs) are adjusted for age, sex, race and ethnicity, education level, income level, marriage status, family history of cardiovascular disease, diagnosis of atrial fibrillation, heart failure, or cancer (excluding nonmelanoma skin cancer) at or before baseline, duration since ASCVD diagnosis, and use of cardiovascular medications. IRR indicates incidence rate ratio; LE8, Life’s Essential 8; PY, person-year.

Among 279 868 veterans without a history of ASCVD at baseline, 12 562 (4.5%) developed MACE during follow-up, with a crude incidence rate of 0.7 per 100 person-years. Among the 133 184 veterans with a history of ASCVD at baseline, 31 212 (23.8%) developed new or recurrent MACE, with a crude incidence rate of 4.2 per 100 person-years (Figure 1). Participants with ASCVD at baseline had significantly greater hazard of MACE compared with those without baseline ASCVD (aHR, 3.87 [95% CI, 3.79-3.96]).

Compared with veterans with baseline ASCVD and low CVH, participants with baseline ASCVD and high CVH had lower hazard of MACE (aHR, 0.52 [95% CI, 0.48-0.56]), and there was a graded decrease in aHR with higher LE8 score (Figure 1). Having no ASCVD at baseline and higher LE8 jointly attenuated the risk of MACE; compared with participants with ASCVD at baseline and low CVH, the multivariate-adjusted hazard for MACE was significantly lower for participants without ASCVD and high CVH (aHR, 0.14 [95% CI, 0.13-0.15]) (Figure 1).

**Figure 2.**
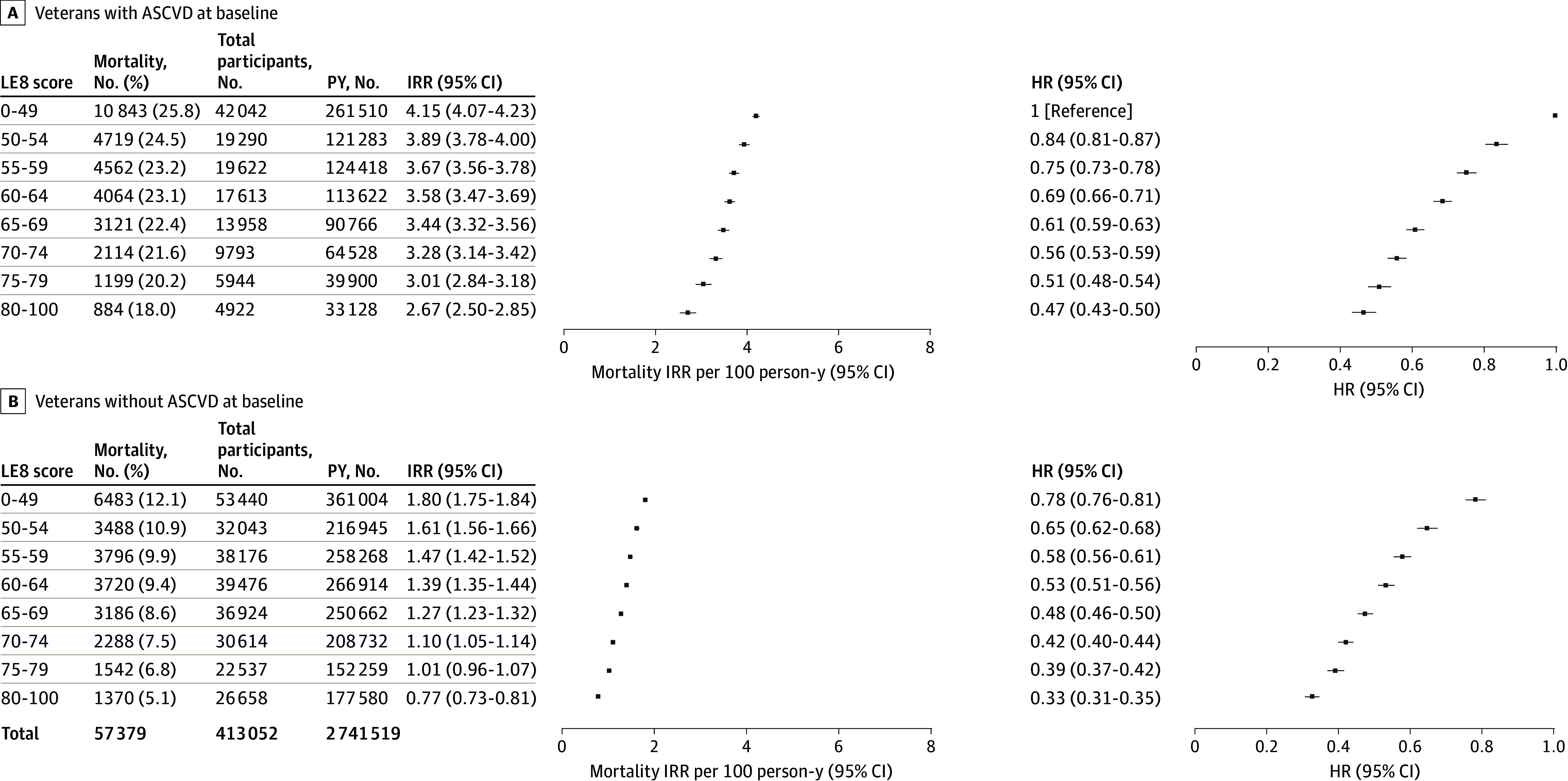
Incident Rates and Hazard of All-Cause Mortality Among Veterans With and Without Atherosclerotic Cardiovascular Disease (ASCVD) at Baseline Hazard ratios (HRs) are adjusted for age, sex, race and ethnicity, education level, income level, marriage status, family history of cardiovascular disease, and diagnosis of atrial fibrillation, heart failure, or cancer (excluding nonmelanoma skin cancer) at or before baseline. IRR indicates incidence rate ratio; LE8, Life’s Essential 8; PY, person-year.

Among 279 868 veterans without a history of ASCVD at baseline, 25 873 (9.2%) deaths were recorded during follow-up, with a crude incidence rate of 1.4 per 100 person-years. Among 133 184 veterans with a history of ASCVD at baseline, 31 506 (23.7%) deaths were recorded, with a crude incidence rate of 3.7 per 100 person-years (Figure 2). The hazard for total mortality risk in participants with ASCVD at baseline was significantly greater compared with those without ASCVD at baseline (aHR, 1.51 [95% CI, 1.48-1.54]). Compared with participants with ASCVD at baseline and low CVH, the hazard of all-cause mortality was lower for participants with ASCVD at baseline and high CVH (aHR, 0.47 [95% CI, 0.43-0.50) and for participants without ASCVD at baseline and high CVH (aHR, 0.33 [95% CI, 0.31-0.35) (Figure 2).

### Sensitivity Analyses

**Figure 3.**
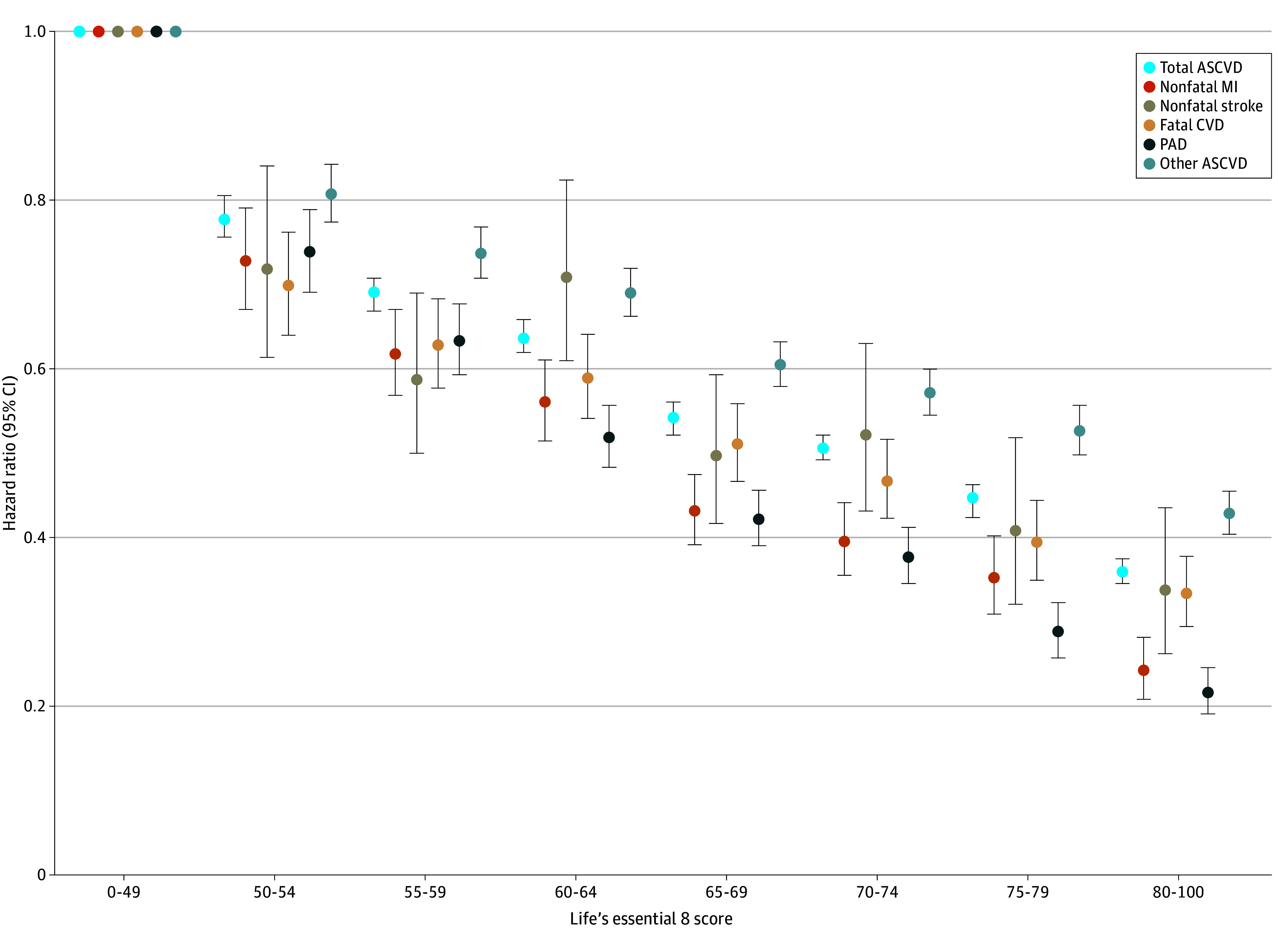
Hazard of Adverse Cardiovascular Outcomes by Life’s Essential 8 Score Hazard ratios are adjusted for age, sex, race and ethnicity, education level, income level, marriage status, family history of cardiovascular disease, and diagnosis of atrial fibrillation, heart failure, or cancer (excluding nonmelanoma skin cancer) at or before baseline. The lowest score group (0-49) was the reference group. ASCVD indicates atherosclerotic cardiovascular disease; CVD, cardiovascular death; MI, myocardial infarction; PAD, peripheral arterial disease.

Sensitivity analyses did not change the main findings, and the association between LE8 score and ASCVD remained significant after restricting participants to only those with complete data on all 8 LE8 metrics; applying a 1-year lag-analysis; excluding veterans with baseline cancer, heart failure, or atrial fibrillation; excluding participants potentially lost-to-follow-up; and among all participants with any LE8 metrics (eTable 4 in Supplement 1). When we examined the associations between LE8 score and subtypes of ASCVD, we found a significant and graded decrease in risk for all subtypes of ASCVD with higher LE8 score (Figure 3). After considering competing risks across different subtypes, each 10-point increase in LE8 score was associated with decreased risk of stroke (aHR, 0.74 [95% CI, 0.71-0.77]), MI (aHR, 0.68 [95% CI, 0.67-0.70]), fatal CVD (aHR, 0.82 [95% CI, 0.81-0.84]), PAD (aHR, 0.69 [95% CI, 0.68-0.70]), and other ASCVD (aHR, 0.83 [95% CI, 0.82-0.84]).

## Discussion

In this large prospective cohort study with 2.7 million person-years of follow-up among 413 052 US veterans, we observed that the association between ASCVD risk and LE8 score was continuous and graded. The protective association between optimal LE8 and incidence of MACE was seen in veterans with and without a history of ASCVD at baseline.

Veterans with baseline ASCVD had a significantly higher absolute risk of developing MACE during the follow-up period compared with veterans without ASCVD at baseline, consistent with previous reports.^4,5,6,7,8^ These findings emphasize that even with optimal adherence to LE8, those with prior ASCVD still had higher relative risk of MACE and all-cause mortality compared with those without ASCVD and suboptimal adherence to LE8, which further stresses the importance of primary prevention. We found that among veterans with ASCVD, the absolute incidence rate of MACE was attenuated from 5.25% among those with low CVH to 2.48% among those with high CVH, and that the potential risk of MACE among these participants may be attenuated by 48% if their LE8 score improved from low to high.

Given the observational nature of this study, results cannot be interpreted as a causal association; however, our estimations are supported by the data from clinical trials. For example, a meta-analysis of 14 randomized clinical trials indicated that structured lifestyle interventions in individuals with established coronary artery disease were associated with reducing the relative risk of fatal cardiovascular events by 18%.^40^ Compared with recent publications focused on primary prevention of ASCVD events with adoption of LE8,^11,12,13^ our findings further provide evidence that not only are both primary and secondary prevention with LE8 optimization important, but also the impact of primary prevention, given how much higher the risk of incident MACE was after first ASCVD event, even with optimal LE8.

### Strengths and Limitations

This study has some strengths, including sample size and the richness of repeated measures within the EHR that allowed us to examine different CVD outcomes over time. Comprehensive lifestyle data that covered different aspects of LE8 was another strength that enabled us to estimate not only the independent associations of individual lifestyles factors but also in combination, a major limitation that previously affected estimations of the global burden of disease in different studies.^41^

Our study has some limitations. First, the study population was predominantly non-Hispanic White male veterans, which may limit generalizability. Second, the cohort was overall older, with a high prevalence of chronic conditions, which may weaken the true associations between LE8 and ASCVD. Third, there is potential reverse causation between observed LE8 and ASCVD associations because people might change their lifestyle habits in response to an ASCVD diagnosis. However, our results were consistent between veterans with and without a history of ASCVD at baseline.

## Conclusions

The findings of this cohort study suggest that a healthy lifestyle aimed at promoting ideal cardiovascular health, such as adherence to the American Heart Association’s LE8is associated with lower risk of ASCVD, as well as lower risk of major adverse events after diagnosis of ASCVD. In addition to optimizing clinical outcomes with support from health care practitioners, the adoption and promotion of lifestyle modifications can be an effective approach for individuals to independently reduce the potential burden of CVD on their health and well-being, and future studies can determine how to best incorporate these important lifestyle factors to help guide CVD prevention and disease management plans.

## Notes

Supplement 1. **eTable 1.** Definition and Scoring Approach to Calculate Life’s Essential 8 Score **eTable 2.** Classification of International Classification of Diseases codes and Current Procedural Terminology Codes for Atherosclerotic Cardiovascular Disease and Its Subtypes **eTable 3.** Comparison of Baseline Characteristics Between Responders and Nonresponders of the Million Veteran Program Lifestyle Survey **eTable 4.** Sensitivity Analyses for the Association Between Life’s Essential 8 Score and Risk of Atherosclerotic Cardiovascular Disease **eAppendix.** Acknowledgement List

Supplement 2.
Data Sharing Statement
