# Supplementary material for: Cardiovascular Health Score and Atherosclerotic Cardiovascular Disease in the Million Veteran Program
Source: JAMA Netw Open. 2024 Dec 6;7(12):e2447902. doi: 10.1001/jamanetworkopen.2024.47902 (PMC11624584; doi:10.1001/jamanetworkopen.2024.47902)
Supplement: Supplement 1. — eTable 1. Definition and Scoring Approach to Calculate Life’s Essential 8 Score eTable 2. Classification of International Classification of Diseases codes and Current Procedural Terminology Codes for Atherosclerotic Cardiovascular Disease and Its Subtypes eTable 3. Comparison of Baseline Characteristics Between Responders and Nonresponders of the Million Veteran Program Lifestyle Survey eTable 4. Sensitivity Analyses for the Association Between Life’s Essential 8 Score and Risk of Atherosclerotic Cardiovascular Disease eAppendix. Acknowledgement List [file jamanetwopen-e2447902-s001.pdf]

Supplemental Online Content

Nguyen XT, Li Y, Gong Y, et al; for the VA Million Veteran Program. Cardiovascular health score and atherosclerotic cardiovascular disease in the Million Veteran Program. *JAMA Netw Open*. 2024;7(12):e2447902. doi:10.1001/jamanetworkopen.2024.47902

eTable 1. Definition and Scoring Approach to Calculate Life's Essential 8 Score

eTable 2. Classification of International Classification of Diseases codes and Current Procedural Terminology Codes for Atherosclerotic Cardiovascular Disease and Its Subtypes

eTable 3. Comparison of Baseline Characteristics Between Responders and Nonresponders of the Million Veteran Program Lifestyle Survey

eTable 4. Sensitivity Analyses for the Association Between Life's Essential 8 Score and Risk of Atherosclerotic Cardiovascular Disease

eAppendix. Acknowledgement List

This supplemental material has been provided by the authors to give readers additional information about their work.

© 2024 Nguyen XT et al. *JAMA Network Open.*

Supplemental Table S1. Definition and scoring approach to calculate Life's Essential 8 (LE8) score

Domain	Metric	Measurement	Quantification and Scoring of LE8
Health Behaviors	Diet	DASH-style diet adherence	**Metric:** DASH-style diet adherence<br>**Scoring (Population):**<br>Points Quantile<br>100 ≥95th percentile (top/ideal diet)<br>80 75th – 94th percentile<br>50 50th – 74th percentile<br>25 25th – 49th percentile<br>0 1st – 24th percentile (bottom/least ideal quartile)
	Physical activity	Self-reported minutes of moderate or vigorous physical activity per week	**Metric:** Minutes of moderate (or greater) intensity activity per week<br>**Scoring:**<br>Points Minutes<br>100 ≥150<br>90 120 – 149<br>80 90 – 119<br>60 60 – 89<br>40 30 – 59<br>20 1 – 29<br>0 0
	Nicotine exposure	Self-reported use of cigarettes	**Metric:** Combustible tobacco use; or secondhand smoke exposure<br>**Scoring:**<br>Points Status<br>100 Never smoker<br>75 Former smoker, ever smoking <15 years<br>50 Former smoker, ever smoking 15-29 years<br>25 Former smoker, ever smoking 30+ years<br>0 Current smoker<br>Subtract 20 points (unless score is 0) for living with active indoor smoker in home
	Sleep health	Self-reported average hours of sleep per night	**Metric:** Average hours of sleep per night<br>**Scoring:**<br>Points Level<br>100 7 – <9<br>90 9 – <10<br>70 6 – <7<br>40 5 – <6 or ≥10<br>20 4 – <5<br>0 <4

© 2024 Nguyen XT et al. *JAMA Network Open.*

Supplemental Table S1 (continued)

Domain	Metric	Measurement	Quantification and Scoring of LE8
Health Factors	Body mass index	Body weight(kg) divided by height squared(m^2)	**Metric:** Body mass index(kg/m^2)<br>**Scoring:**<br><u>Points Level</u><br>100 <25<br>70 25.0 – 29.9<br>30 30.0 – 34.9<br>15 35.0 – 39.9<br>0 ≥40.0
	Blood lipids	Plasma total and HDL-cholesterol with calculation of non-HDL-cholesterol	**Metric:** Non-HDL-cholesterol(mg/dL)<br>**Scoring:**<br><u>Points Level</u><br>100 <130<br>60 130 – 159<br>40 160 – 189<br>20 190 – 219<br>0 ≥220<br><br>If drug-treated level, subtract 20 points
	Blood glucose	Hemoglobin A1c and diagnosis of diabetes	**Metric:** Hemoglobin A1c (%)<br>**Scoring:**<br><u>Points Level</u><br>100 No history of diabetes and HbA1c < 5.7<br>60 No diabetes and HbA1c 5.7-6.4<br>40 Diabetes with HbA1c <7.0<br>30 Diabetes with HbA1c 7.0 – 7.9<br>20 Diabetes with HbA1c 8.0 – 8.9<br>10 Diabetes with Hb A1c 9.0 – 9.9<br>0 Diabetes with HbA1c ≥10.0
	Blood pressure	Measured systolic and diastolic blood pressure	**Metric:** Systolic and diastolic blood pressure (mm Hg)<br>**Scoring:**<br><u>Points Level</u><br>100 <120/<80<br>75 120-129/<80<br>50 130-139 or 80-89<br>25 140-159 or 90-99<br>0 ≥160 or ≥100<br><br>Subtract 20 points if treated level

© 2024 Nguyen XT et al. *JAMA Network Open.*

Supplemental Table S2: Classification of International Classification of Diseases (ICD) codes and Current Procedural Terminology (CPT) codes for atherosclerotic cardiovascular disease (ASCVD) and its subtypes

ASCVD subtypes	ICD and CPT Codes
**(1) Myocardial Infarction**	
ICD 9	410.x, 411.x, 412.x (for baseline exclusion only)
ICD 10	I21.x, I22.x, I25.2 (for baseline exclusion only), I23.x
**(2) Non-MI Ischemic Heart Disease (IHD)**	
ICD 9	410.0, 410.00, 410.01, 410.02, 410.1, 410.10, 410.11, 410.12, 410.2, 410.20, 410.21, 410.22, 410.3, 410.30, 410.31, 410.32, 410.4, 410.40, 410.41, 410.42, 410.5, 410.50, 410.51, 410.52, 410.6, 410.60, 410.61, 410.62, 410.7, 410.70, 410.71, 410.72, 410.8, 410.80, 410.81, 410.82, 410.9, 410.90, 410.91, 410.92, 411.0, 411.1, 411.8, 411.81, 411.89, 412, 413.0, 413.1, 413.9, 414.0, 414.00, 414.01, 414.02, 414.03, 414.04, 414.05, 414.06, 414.07, 414.10, 414.11, 414.12, 414.19, 414.2, 414.3, 414.4, 414.8, 414.9
ICD 10	I20.0, I20.1, I20.2, I20.8, I20.9, I21.01, I21.02, I21.09, I21.11, I21.19, I21.21, I21.29, I21.3, I21.4, I21.9, I21.A1, I21.A9, I22.0, I22.1, I22.2, I22.8, I22.9, I23.0, I23.1, I23.2, I23.3, I23.4, I23.5, I23.6, I23.7, I23.8, I24.0, I24.1, I24.8, I24.9, I29.A1, I29.A9, I25.10, I25.2, I25.110, I25.111, I25.112, I25.118, I25.119, I25.3, I25.41, I25.42, I25.5, I25.6, I25.700, I25.701, I25.702, I25.708, I25.709, I25.710, I25.711, I25.712, I25.718, I25.719, I25.720, I25.721, I25.722, I25.728, I25.729, I25.730, I25.731, I25.732, I25.738, I25.739, I25.750, I25.751, I25.752, I25.758, I25.759, I25.760, I25.761, I25.762, I25.768, I25.769, I25.790, I25.791, I25.792, I25.798, I25.799, I25.810, I25.811, I25.812, I25.82, I25.83, I25.84, I25.89, I25.9

© 2024 Nguyen XT et al. *JAMA Network Open.*

Supplemental Table S2 (continued):

ASCVD subtypes	ICD and CPT Codes
**(2) Non-MI Ischemic Heart Disease (IHD)**	
CPT/ICD procedures	33140, 33510, 33511, 33512, 33513, 33514, 33516, 33517, 33518, 33519, 33520, 33521, 33522, 33523, 33530, 33533, 33534, 33535, 33535, 33536, 33572, 92920, 92921, 92924, 92925, 92928, 92929, 92933, 92934, 92937, 92938, 92941, 92943, 92944, 92980, 92981, 92982, 92984, 92995, 92996, 93540, 93564, 93570, C9600, C9601, C9602, C9603, C9604, C9605, C9606, C9607, C9608, S2205, S2206, S2207, S2208, S2209, 0.66, 36.01, 36.02, 36.03, 36.04, 36.05, 36.06, 36.07, 36.09, 36.1, 36.11, 36.11, 36.12, 36.13, 36.14, 36.15, 36.16, 36.17, 36.19, 36.2, 36.31, 36.32, 36.39, 36.99, 021008C, 210098, 021009C, 02100A3, 02100AC, 02100AW, 02100AW, 02100J8, 02100J9, 02100KF, 02100KF, 02100Z9, 021049F, 02104A8, 02104AF, 02104J8, 02104JC, 02104JW, 02104K8, 02104KC, 02104KF, 02104Z9, 02104ZC, 021109C, 021109C, 021109W, 02110A9, 02110J9, 02110JC, 02110JW, 02110K8, 021148W, 02114A8, 02114AC, 02114J8, 02114J9, 02114JC, 02114KW, 02114Z8, 02114ZC, 021208C, 021209C, 02120AC, 02120AW, 02120JC, 02120KC, 02120KC, 021249W, 02124KC, 02124ZC, 02124ZC, 021309W, 02130JC, 02130JC, 021008F, 210093, 210098, 021009C, 02100A3, 02100A9, 02100A9, 02100AC, 02100J9, 02100JF, 02100JF, 02100K9, 02100KC, 02100KC, 02100KW, 02100KW, 02100Z3, 02100ZC, 02100ZF, 021049F, 02104A8, 02104A9, 02104AC, 02104J8, 02104J9, 02104JC, 02104JW, 02104KC, 02104Z3, 02104Z8, 02104ZF, 02104ZF, 021108C, 021108W, 021109W, 02110A8, 02110AC, 02110AW, 02110J8, 02110JC, 02110KC, 02110KW, 02110Z9, 02110Z9, 02114A8, 02114A9, 02114AC, 02114AW, 02114AW, 02114J8, 02114J9, 02114JC, 02114JW, 02114K9, 02114Z9, 02114ZC, 021008W, 210093, 021009F, 02100A8, 02100J3, 02100J3, 02100J8, 02100JC, 02100K3, 02100Z3, 02100Z8, 02100ZC, 021048C, 021048W, 021049W, 02104A3, 02104A3, 02104AC, 02104AW, 02104AW, 02104J3, 02104JF, 02104K3, 02104K3, 02104K8, 02104K9, 02104KW, 02104KW, 02104Z8, 02104ZC, 02110A8, 02110J9, 02110K9, 02110Z8, 02110ZC, 02110ZC, 021149C, 021149C, 021149W, 02114A9, 02114JW, 02114K8, 02114K8, 02114K9, 02114KC, 02114KC, 02114KW, 021209C, 02120JC, 02120JW, 02120JW, 02120KW, 02120Z9, 02120ZC, 021249C, 210099, 210099, 021009F, 021009W, 021009W, 02100A8, 02100AF, 02100AF, 02100JC, 02100JW, 02100JW, 02100K3, 02100K8, 02100K8, 02100K9, 02100Z8, 02100Z9, 02100ZF, 021048F, 021049C, 021049C, 021049W, 02104A9, 02104AF, 02104J3, 02104J9, 02104JF, 02104K9, 02104KF, 02104Z3, 02104Z9, 02110A9, 02110AC, 02110AW, 02110J8, 02110K8, 02110K9, 02110KC, 02110KW, 02110Z8, 021148C, 021149W, 02114Z8, 02114Z9, 021209W, 02120AC, 021248W, 02124JC, 02124JW, 021308C, 021309W, 02130AC, 02130KW, 021348C, 021349C, 021349W, 02130JW, 02130JW, 02130KW, 02134AC, 02134JC, 02134JC, 02134JW, 02134ZC, 02713ZZ, 02713ZZ, 02724ZZ, 06B04ZZ, 02134AC, 02134KC, 02134KC, 02134KW, 02703ZZ, 02704ZZ, 02714ZZ, 02723ZZ, 02733ZZ, 02734ZZ, 03BB0ZZ, 021249C, 02124AW, 02124AW, 02124JW, 02124KC, 02124KW, 021308W, 02130AC, 02130AW, 02130AW, 02130ZC, 021348W, 02134JW, 02134KW, 02703ZZ, 02724ZZ, 02733ZZ, 021208W, 021209W, 02120AW, 02120KW, 02120Z9, 02120ZC, 021248C, 021249W, 02124AC, 02124AC, 02124JC, 02124KW, 021309C, 021309C, 02130KC, 02130KC, 02130ZC, 021349C, 021349W, 02134AW, 02134AW, 02134ZC, 02704ZZ, 02714ZZ, 02723ZZ, 02734ZZ
**(3) Stroke**	
ICD 9	430.x, 431.x, 436.x, 433.01, 433.11, 433.21, 433.31, 433.81, 433.91, 434.0, 434.00, 434.1, 434.10, 434.9, 434.90, 437.0, 437.6
ICD 10	I60.x, I61.x, I63.019, I63.039, I63.119, I63.139, I63.20, I63.219, I63.22, I63.239, I63.30, I63.319, I63.329, I63.339, I63.349, I63.40, I63.419, I63.429, I63.439, I63.449, I63.50, I63.519, I63.529, I63.539, I63.549, I63.59

© 2024 Nguyen XT et al. *JAMA Network Open.*

Supplemental Table S2 (continued):

ASCVD subtypes	ICD and CPT Codes
**(4) Peripheral Arterial Disease (PAD)**	
ICD 9	440.1, 440.2, 440.20, 440.21, 440.22, 440.23, 440.24, 440.29, 440.30, 440.31, 440.32, 440.8, 440.9, 443.9, 444.0, 444.1, 444.21, 444.22, 444.81, 444.89, 444.9, 445.01, 445.02, 445.81, 445.89
ICD 10	I70.1, I70.209, I70.219, I70.229, I70.25, I70.269, I70.399, I70.499, I70.599, I70.8, I70.90, I70.91, I73.9, I74.11, I74.2, I74.3, I74.5, I74.8, I74.9, I75.019, I75.029, I75.81, I75.89
CPT/ICD procedures	84.10, 84.11, 84.12, 84.14, 84.15, 84.17, 84.9, 38.18, 38.08, 38.38, 38.48, 39.50, 39.25, 39.29, 39.90, 39.49, 39.56, 39.57, 39.58, 27295, 27590, 27591, 27592, 27598, 27880, 27881, 27882, 28800, 28805, 35302, 35303, 35304, 35305, 35306, 35331, 35351, 35355, 35361, 35363, 35371, 35372, 35381, 35452, 35454, 35456, 35459, 35470, 35472, 35473, 35474, 35481, 35482, 35483, 35485, 35491, 35492, 35493, 35495, 35521, 35533, 35537, 35538, 35539, 35540, 35541, 35546, 35548, 35549, 35551, 35556, 35558, 35563, 35565, 35566, 35571, 35582, 35583, 35585, 35587, 35621, 35623, 35637, 35638, 35641, 35646, 35647, 35651, 35654, 35656, 35661, 35663, 35665, 35666, 35671, 37184, 37185, 37186, 37205, 37206, 37207, 37208, 35226, 35256, 35286, 35700, 35721, 35741, 35876, 35879, 35881, 35883, 35884
**(5) Other ASCVD**	
ICD 9	433.0, 433.00, 433.1, 433.10, 433.2, 433.20, 433.3, 433.30, 433.8, 433.80, 433.9, 433.90, 434.01, 434.11, 434.91, 435.0, 435.1, 435.2, 435.3, 435.8, 435.9, 781.4, V12.54
ICD 10	I63.01, I63.129, I65.09, I65.1, I65.29, I65.8, I65.9, I66.09, I66.19, I66.29, I66.9, H34.00, H34.13, H34.234, H34.219, H34.9, H53.129, G45.0, G45.8, G45.1, G45.8, G45.9, I67.848, I67.89, R29.5, Z86.73
CPT/ICD procedures	00.61, 00.63, 38.12
**(6) CVD death**	
ICD 10	I (0, 1, 2, 5, 6, 7, 8, 9, 10, 11, 12, 13, 20, 21, 22, 24, 25, 26, 27, 28, 30, 31, 32, 33, 34, 35, 36, 37, 38, 40, 42, 44, 45, 46, 47, 48, 49, 50, 51, 60, 61, 62, 63, 67, 68, 69, 70, 71, 72, 73, 74, 77, 78, 80, 81, 82, 83, 84, 85, 86, 87, 89, 95, 97, 99), M30, M31, R0, R58, G45

© 2024 Nguyen XT et al. *JAMA Network Open.*

Supplemental Table S3. Comparison of baseline characteristics between responders and non-responders of the MVP Lifestyle Survey[a,b]

Characteristic, N (%)	MVP participants enrolled by September 2023			
	Lifestyle Responders		Lifestyle non-responders	All
	Study Population N=413,052	Excluded[b] N=4,675	N=495,592	N=913,319
Age, years (SD)	65.8 (12.1)	71.8 (12.7)	57.5 (15.1)	61.3 (14.4)
Female,	34,890 (8.4%)	257 (5.5%)	57,012 (11.5%)	92,159 (10.1%)
Race and ethnicity,				
Hispanic	26,571 (6.4%)	218 (4.7%)	48,015 (9.7%)	74,804 (8.2%)
Non-Hispanic Black	44,777 (10.8%)	467 (10.0%)	116,012 (23.4%)	161,256 (17.7%)
Non-Hispanic White	329,365 (79.7%)	3,674 (78.6%)	298,595 (60.3%)	631,634 (69.2%)
Other[c]	12,339 (3.0%)	316 (6.8%)	32,970 (6.7%)	45,625 (5.0%)
Diseases at/before baseline				
Atrial fibrillation	42,224 (10.2%)	525 (11.2%)	37,770 (7.6%)	80,519 (8.8%)
Heart failure	32,470 (7.9%)	447 (9.6%)	38,491 (7.8%)	71,408 (7.8%)
Cancer	221,072 (53.5%)	1,576 (33.7%)	229,558 (46.3%)	452,206 (49.5%)

[a]Values are means or percentages and are standardized to the age distribution of the study population except age.

[b]Those who responded to Lifestyle Questionnaires but with implausible death records and/or with missing data on 3 or more LE8 factors were excluded from the study population.

[c]Other includes participants who responded "No, not Spanish, Hispanic, Latino" and any combination of the following: American Indian/Alaska Native, Chinese, Japanese, Asian Indian, Other Asian, Filipino, Pacific Islander, Other"

© 2024 Nguyen XT et al. *JAMA Network Open.*

Supplemental Table S4. Sensitivity Analyses for the Association Between Life's Essential 8 (LE8) Score and Risk of ASCVD[a]

	Sensitivity 1	Sensitivity 2	Sensitivity 3	Sensitivity 4	Sensitivity 5
Characteristic	Full data on eight LE8 metrics	Excluded participants with <1 year follow-up	Excluded cancers, heart failure, and atrial fibrillation at baseline	Excluded no medical visit after baseline	Using all data on eight LE8 metrics – no missing exclusion
Study population	212,006	269,464	140,554	268,121	282,947
No. of ASCVD	35,727 (16.9%)	37,000 (13.7%)	17,365 (12.4%)	45,067 (16.8%)	45,411 (16.0%)
LE8 score					
<50	1.0 (ref.)	1.0 (ref.)	1.0 (ref.)	1.0 (ref.)	1.0 (ref.)
50-	0.78 (0.76- 0.81)	0.80 (0.77- 0.82)	0.75 (0.71- 0.79)	0.79 (0.77- 0.82)	0.79 (0.77- 0.82)
55-	0.69 (0.67- 0.71)	0.70 (0.68- 0.73)	0.69 (0.66- 0.73)	0.70 (0.68- 0.72)	0.70 (0.68- 0.73)
60-	0.64 (0.61- 0.66)	0.65 (0.63- 0.67)	0.62 (0.59- 0.66)	0.65 (0.63- 0.67)	0.65 (0.63- 0.67)
65-	0.55 (0.53- 0.57)	0.56 (0.54- 0.58)	0.51 (0.48- 0.54)	0.55 (0.54- 0.57)	0.55 (0.53- 0.57)
70-	0.50 (0.48- 0.52)	0.52 (0.50- 0.54)	0.47 (0.44- 0.50)	0.52 (0.50- 0.54)	0.52 (0.50- 0.53)
75-	0.44 (0.42- 0.46)	0.46 (0.44- 0.48)	0.42 (0.39- 0.45)	0.46 (0.44- 0.48)	0.45 (0.44- 0.48)
80-100	0.36 (0.35- 0.38)	0.37 (0.35- 0.39)	0.32 (0.30- 0.35)	0.38 (0.36- 0.39)	0.37 (0.35- 0.38)
P for trend	<0.001	<0.001	<0.001	<0.001	<0.001
HR per 10 score	0.78 (0.77- 0.79)	0.79 (0.78- 0.79)	0.76 (0.75- 0.77)	0.79 (0.78- 0.79)	0.78 (0.78- 0.79)

[a]HRs were calculated using a Cox proportional hazards regression model adjusted for age (continuous), sex (male or female), race/ethnicity (Hispanic, non-Hispanic Black, non-Hispanic White, and other), education level (⩽ high school or General Educational Development(GED), some college, or college or above), income level (< $30,000, $30,000-$59,000, ⩾ $60,000 or missing), marital status (currently married: yes or no or missing), family history of cardiovascular disease (yes or no), diagnosis of atrial fibrillation, heart failure or cancer (excluding non-melanoma skin cancer) at or before baseline.

© 2024 Nguyen XT et al. *JAMA Network Open.*

Supplemental: Acknowledgement List

VA Million Veteran Program:

MVP Program Office

- Program Director - Sumitra Muralidhar, Ph.D.
  US Department of Veterans Affairs, 810 Vermont Avenue NW, Washington, DC 20420
- Associate Director, Scientific Programs - Jennifer Moser, Ph.D.
  US Department of Veterans Affairs, 810 Vermont Avenue NW, Washington, DC 20420
- Associate Director, Cohort Management & Public Relations - Jennifer E. Deen, B.S.
  US Department of Veterans Affairs, 810 Vermont Avenue NW, Washington, DC 20420

MVP Executive Committee

- Co-Chair: J. Michael Gaziano, M.D., M.P.H.
  VA Boston Healthcare System, 150 S. Huntington Avenue, Boston, MA 02130
- Co-Chair: Sumitra Muralidhar, Ph.D.
  US Department of Veterans Affairs, 810 Vermont Avenue NW, Washington, DC 20420
- Jean Beckham, Ph.D.
  Durham VA Medical Center, 508 Fulton Street, Durham, NC 27705
- Kyong-Mi Chang, M.D.
  Philadelphia VA Medical Center, 3900 Woodland Avenue, Philadelphia, PA 19104
- Philip S. Tsao, Ph.D.
  VA Palo Alto Health Care System, 3801 Miranda Avenue, Palo Alto, CA 94304
- Shiuh-Wen Luoh, M.D., Ph.D.
  VA Portland Health Care System, 3710 SW US Veterans Hospital Rd, Portland, OR 97239
  US Department of Veterans Affairs, 810 Vermont Avenue NW, Washington, DC 20420
- Juan P. Casas, M.D., Ph.D., Ex-Officio
  VA Boston Healthcare System, 150 S. Huntington Avenue, Boston, MA 02130

MVP Principal Investigators

- J. Michael Gaziano, M.D., M.P.H.
  VA Boston Healthcare System, 150 S. Huntington Avenue, Boston, MA 02130
- Philip S. Tsao, Ph.D.
  VA Palo Alto Health Care System, 3801 Miranda Avenue, Palo Alto, CA 94304

MVP Operations

- MVP Executive Director – Juan P. Casas, M.D., Ph.D.
  VA Boston Healthcare System, 150 S. Huntington Avenue, Boston, MA 02130
- Director of Regulatory Affairs – Lori Churby, B.S.
  VA Palo Alto Health Care System, 3801 Miranda Avenue, Palo Alto, CA 94304
- MVP Cohort Management Director – Stacey B. Whitbourne, Ph.D.
  VA Boston Healthcare System, 150 S. Huntington Avenue, Boston, MA 02130
- MVP Recruitment/Enrollment Director - Jessica V. Brewer, M.P.H.
  VA Boston Healthcare System, 150 S. Huntington Avenue, Boston, MA 02130
- Director, VA Central Biorepository, Boston – Mary T. Brophy M.D., M.P.H.
  VA Boston Healthcare System, 150 S. Huntington Avenue, Boston, MA 02130
- Executive Director for MVP Biorepositories - Luis E. Selva, Ph.D.
  VA Boston Healthcare System, 150 S. Huntington Avenue, Boston, MA 02130

© 2024 Nguyen XT et al. *JAMA Network Open.*

- MVP Informatics, Boston – Shahpoor (Alex) Shayan, M.S.
  VA Boston Healthcare System, 150 S. Huntington Avenue, Boston, MA 02130
- Director, MVP Data Operations/Analytics, Boston – Kelly Cho, M.P.H., Ph.D.
  VA Boston Healthcare System, 150 S. Huntington Avenue, Boston, MA 02130
- Director, Center for Computational and Data Science (C-DACS) & Genomics Core – Saiju Pyarajan Ph.D.
  VA Boston Healthcare System, 150 S. Huntington Avenue, Boston, MA 02130
- Director, Molecular Data Core – Philip S. Tsao, Ph.D.
  VA Palo Alto Health Care System, 3801 Miranda Avenue, Palo Alto, CA 94304
- Director, Phenomics Data Core – Kelly Cho, M.P.H, Ph.D.
  VA Boston Healthcare System, 150 S. Huntington Avenue, Boston, MA 02130
- Director, VA Informatics and Computing Infrastructure (VINCI) – Scott L. DuVall, Ph.D.
  VA Salt Lake City Health Care System, 500 Foothill Drive, Salt Lake City, UT 84148
- MVP Coordinating Centers
  - Cooperative Studies Program Clinical Research Pharmacy Coordinating Center, Albuquerque – Todd Connor, Pharm.D.; Dean P. Argyres, B.S., M.S.
    New Mexico VA Health Care System, 1501 San Pedro Drive SE, Albuquerque, NM 87108
  - Genomics Coordinating Center, Palo Alto – Philip S. Tsao, Ph.D.
    VA Palo Alto Health Care System, 3801 Miranda Avenue, Palo Alto, CA 94304
  - MVP Boston Coordinating Center, Boston - J. Michael Gaziano, M.D., M.P.H.
    VA Boston Healthcare System, 150 S. Huntington Avenue, Boston, MA 02130
  - MVP Information Center, Canandaigua – Brady Stephens, M.S.
    Canandaigua VA Medical Center, 400 Fort Hill Avenue, Canandaigua, NY 14424

Current MVP Local Site Investigators

- Atlanta VA Medical Center (Peter Wilson, M.D.)
  1670 Clairmont Road, Decatur, GA 30033
- Bay Pines VA Healthcare System (Rachel McArdle, Ph.D.)
  10,000 Bay Pines Blvd Bay Pines, FL 33744
- Birmingham VA Medical Center (Louis Dellitalia, M.D.)
  700 S. 19th Street, Birmingham AL 35233
- Central Western Massachusetts Healthcare System (Kristin Mattocks, Ph.D., M.P.H.)
  421 North Main Street, Leeds, MA 01053
- Cincinnati VA Medical Center (John Harley, M.D., Ph.D.)
  3200 Vine Street, Cincinnati, OH 45220
- Clement J. Zablocki VA Medical Center (Jeffrey Whittle, M.D., M.P.H.)
  5000 West National Avenue, Milwaukee, WI 53295
- VA Northeast Ohio Healthcare System (Frank Jacono, M.D.)
  10701 East Boulevard, Cleveland, OH 44106
- Durham VA Medical Center (Jean Beckham, Ph.D.)
  508 Fulton Street, Durham, NC 27705
- Edith Nourse Rogers Memorial Veterans Hospital (John Wells., Ph.D.)
  200 Springs Road, Bedford, MA 01730
- Edward Hines, Jr. VA Medical Center (Salvador Gutierrez, M.D.)
  5000 South 5th Avenue, Hines, IL 60141
- Veterans Health Care System of the Ozarks (Kathrina Alexander, M.D.)
  1100 North College Avenue, Fayetteville, AR 72703
- Fargo VA Health Care System (Kimberly Hammer, Ph.D.)
  2101 N. Elm, Fargo, ND 58102

© 2024 Nguyen XT et al. *JAMA Network Open.*

- VA Health Care Upstate New York (James Norton, Ph.D.)
  113 Holland Avenue, Albany, NY 12208
- New Mexico VA Health Care System (Gerardo Villareal, M.D.)
  1501 San Pedro Drive, S.E. Albuquerque, NM 87108
- VA Boston Healthcare System (Scott Kinlay, M.B.B.S., Ph.D.)
  150 S. Huntington Avenue, Boston, MA 02130
- VA Western New York Healthcare System (Junzhe Xu, M.D.)
  3495 Bailey Avenue, Buffalo, NY 14215-1199
- Ralph H. Johnson VA Medical Center (Mark Hamner, M.D.)
  109 Bee Street, Mental Health Research, Charleston, SC 29401
- Columbia VA Health Care System (Roy Mathew, M.D.)
  6439 Garners Ferry Road, Columbia, SC 29209
- VA North Texas Health Care System (Sujata Bhushan, M.D.)
  4500 S. Lancaster Road, Dallas, TX 75216
- Hampton VA Medical Center (Pran Iruvanti, D.O., Ph.D.)
  100 Emancipation Drive, Hampton, VA 23667
- Richmond VA Medical Center (Michael Godschalk, M.D.)
  1201 Broad Rock Blvd., Richmond, VA 23249
- Iowa City VA Health Care System (Zuhair Ballas, M.D.)
  601 Highway 6 West, Iowa City, IA 52246-2208
- Eastern Oklahoma VA Health Care System (River Smith, Ph.D.)
  1011 Honor Heights Drive, Muskogee, OK 74401
- James A. Haley Veterans' Hospital (Stephen Mastorides, M.D.)
  13000 Bruce B. Downs Blvd, Tampa, FL 33612
- James H. Quillen VA Medical Center (Jonathan Moorman, M.D., Ph.D.)
  Corner of Lamont & Veterans Way, Mountain Home, TN 37684
- John D. Dingell VA Medical Center (Saib Gappy, M.D.)
  4646 John R Street, Detroit, MI 48201
- Louisville VA Medical Center (Jon Klein, M.D., Ph.D.)
  800 Zorn Avenue, Louisville, KY 40206
- Manchester VA Medical Center (Nora Ratcliffe, M.D.)
  718 Smyth Road, Manchester, NH 03104
- Miami VA Health Care System (Ana Palacio, M.D., M.P.H.)
  1201 NW 16th Street, 11 GRC, Miami FL 33125
- Michael E. DeBakey VA Medical Center (Olaoluwa Okusaga, M.D.)
  2002 Holcombe Blvd, Houston, TX 77030
- Minneapolis VA Health Care System (Maureen Murdoch, M.D., M.P.H.)
  One Veterans Drive, Minneapolis, MN 55417
- N. FL/S. GA Veterans Health System (Peruvemba Sriram, M.D.)
  1601 SW Archer Road, Gainesville, FL 32608
- Northport VA Medical Center (Shing Shing Yeh, Ph.D., M.D.)
  79 Middleville Road, Northport, NY 11768
- Overton Brooks VA Medical Center (Neeraj Tandon, M.D.)
  510 East Stoner Ave, Shreveport, LA 71101
- Philadelphia VA Medical Center (Darshana Jhala, M.D.)
  3900 Woodland Avenue, Philadelphia, PA 19104
- Phoenix VA Health Care System (Samuel Aguayo, M.D.)
  650 E. Indian School Road, Phoenix, AZ 85012
- Portland VA Medical Center (David Cohen, M.D.)
  3710 SW U.S. Veterans Hospital Road, Portland, OR 97239

© 2024 Nguyen XT et al. *JAMA Network Open.*

- Providence VA Medical Center (Satish Sharma, M.D.)
  830 Chalkstone Avenue, Providence, RI 02908
- Richard Roudebush VA Medical Center (Suthat Liangpunsakul, M.D., M.P.H.)
  1481 West 10th Street, Indianapolis, IN 46202
- Salem VA Medical Center (Kris Ann Oursler, M.D.)
  1970 Roanoke Blvd, Salem, VA 24153
- San Francisco VA Health Care System (Mary Whooley, M.D.)
  4150 Clement Street, San Francisco, CA 94121
- South Texas Veterans Health Care System (Sunil Ahuja, M.D.)
  7400 Merton Minter Boulevard, San Antonio, TX 78229
- Southeast Louisiana Veterans Health Care System (Joseph Constans, Ph.D.)
  2400 Canal Street, New Orleans, LA 70119
- Southern Arizona VA Health Care System (Paul Meyer, M.D., Ph.D.)
  3601 S 6th Avenue, Tucson, AZ 85723
- Sioux Falls VA Health Care System (Jennifer Greco, M.D.)
  2501 W 22nd Street, Sioux Falls, SD 57105
- St. Louis VA Health Care System (Michael Rauchman, M.D.)
  915 North Grand Blvd, St. Louis, MO 63106
- Syracuse VA Medical Center (Richard Servatius, Ph.D.)
  800 Irving Avenue, Syracuse, NY 13210
- VA Eastern Kansas Health Care System (Melinda Gaddy, Ph.D.)
  4101 S 4th Street Trafficway, Leavenworth, KS 66048
- VA Greater Los Angeles Health Care System (Agnes Wallbom, M.D., M.S.)
  11301 Wilshire Blvd, Los Angeles, CA 90073
- VA Long Beach Healthcare System (Timothy Morgan, M.D.)
  5901 East 7th Street Long Beach, CA 90822
- VA Maine Healthcare System (Todd Stapley, D.O.)
  1 VA Center, Augusta, ME 04330
- VA New York Harbor Healthcare System (Peter Liang, M.D., M.P.H.)
  423 East 23rd Street, New York, NY 10010
- VA Pacific Islands Health Care System (Daryl Fujii, Ph.D.)
  459 Patterson Rd, Honolulu, HI 96819
- VA Palo Alto Health Care System (Philip Tsao, Ph.D.)
  3801 Miranda Avenue, Palo Alto, CA 94304-1290
- VA Pittsburgh Health Care System (Patrick Strollo, Jr., M.D.)
  University Drive, Pittsburgh, PA 15240
- VA Puget Sound Health Care System (Edward Boyko, M.D.)
  1660 S. Columbian Way, Seattle, WA 98108-1597
- VA Salt Lake City Health Care System (Jessica Walsh, M.D.)
  500 Foothill Drive, Salt Lake City, UT 84148
- VA San Diego Healthcare System (Samir Gupta, M.D., M.S.C.S.)
  3350 La Jolla Village Drive, San Diego, CA 92161
- VA Sierra Nevada Health Care System (Mostaqul Huq, Pharm.D., Ph.D.)
  975 Kirman Avenue, Reno, NV 89502
- VA Southern Nevada Healthcare System (Joseph Fayad, M.D.)
  6900 North Pecos Road, North Las Vegas, NV 89086
- VA Tennessee Valley Healthcare System (Adriana Hung, M.D., M.P.H.)
  1310 24th Avenue, South Nashville, TN 37212
- Washington DC VA Medical Center (Jack Lichy, M.D., Ph.D.)
  50 Irving St, Washington, D. C. 20422

© 2024 Nguyen XT et al. *JAMA Network Open.*

- W.G. (Bill) Hefner VA Medical Center (Robin Hurley, M.D.)
  1601 Brenner Ave, Salisbury, NC 28144
- White River Junction VA Medical Center (Brooks Robey, M.D.)
  163 Veterans Drive, White River Junction, VT 05009
- William S. Middleton Memorial Veterans Hospital (Prakash Balasubramanian, M.D.)
  2500 Overlook Terrace, Madison, WI 53705

© 2024 Nguyen XT et al. *JAMA Network Open.*
