# Supplementary material for: Cardiovascular Health Score and Atherosclerotic Cardiovascular Disease in the Million Veteran Program
Source: JAMA Netw Open. 2024 Dec 6;7(12):e2447902. doi: 10.1001/jamanetworkopen.2024.47902 (PMC11624584; doi:10.1001/jamanetworkopen.2024.47902)
Supplement: Supplement 2. — Data Sharing Statement [file jamanetwopen-e2447902-s002.pdf]

Data Sharing Statement

Nguyen. Cardiovascular Health Score and Atherosclerotic Cardiovascular Disease in the Million Veteran Program. *JAMA Netw Open*. Published December 06, 2024. doi:10.1001/jamanetworkopen.2024.47902

Data
Data available: No

Additional Information
Explanation for why data not available: Data used in this study cannot be shared publicly because of VA policies regarding data privacy and security. Data contain potentially identifying and sensitive patient information. All relevant summary level data are included in the manuscript. For investigators with appropriate authorizations within the Department of Veterans Affairs, requests for data access can be made to the corresponding author.
